# Induction of Systemic Resistance in *Hibiscus sabdariffa* Linn. to Control Root Rot and Wilt Diseases Using Biotic and Abiotic Inducers

**DOI:** 10.3390/biology12060789

**Published:** 2023-05-30

**Authors:** Hamada F. A. Ahmed, Sameh Elnaggar, Gomaa A. Abdel-Wahed, Ragab S. Taha, Awais Ahmad, Wadei A. Al-Selwey, Hoda M. H. Ahmed, Naeem Khan, Mahmoud F. Seleiman

**Affiliations:** 1 Department of Ornamental, Medicinal and Aromatic Plant Diseases, Plant Pathology Research Institute, Agricultural Research Center (ARC), Giza P.O. Box 12619, Egypt; 2 Department of Botany (Plant Pathology), Faculty of Agriculture, Fayoum University, Fayoum P.O. Box 63514, Egypt; 3 Department of Plant Pathology, Faculty of Agriculture, Beni-Suef University, Beni-Suef P.O. Box 62521, Egypt; 4 Department of Plant Production, College of Food and Agriculture Sciences, King Saud University, P.O. Box 2460, Riyadh 11451, Saudi Arabia; 5 Department of Agronomy, Institute of Food and Agricultural Sciences, University of Florida, Gainesville, FL 32611, USA

**Keywords:** systemic resistance, *Hibiscus sabdariffa* L., biotic/abiotic inducers, root rot/wilt disease, enzyme activities, biochemical changes

## Abstract

**Simple Summary:**

Fungal root rot and wilt diseases are among the most urgent obstacles to roselle production as they attack seedlings and mature plants, causing significant yield losses. Outbreaks of such diseases can be prevented with chemical fungicides. Unfortunately, the excessive use of fungicides can pollute the environment and cause harmful effects in humans and animals. Therefore, a number of biotic and abiotic inducers were selected for the induction of systemic resistance (ISR) in roselle. The used inducers have shown a good ability to inhibit the growth of pathogenic fungi *in vitro*, and strongly reduce disease development *in vivo*. With the view that plants can defend themselves through a variety of chemical mechanisms, we estimated the phytochemicals and the activities of defensive enzymes. In conclusion, ISR has become a good target for suppressing roselle root rot and wilt, and promoting crop growth without environmental risks.

**Abstract:**

The possibility of inducing systemic resistance in roselle against root rot and wilt diseases was investigated using biotic and abiotic inducers. The biotic inducers included three biocontrol agents (i.e., *Bacillus subtilis*, *Gliocladium catenulatum*, and *Trichoderma asperellum*) and two biofertilizers (i.e., microbein and mycorrhizeen), while the abiotic inducers included three chemical materials (i.e., ascorbic acid, potassium silicate, and salicylic acid). In addition, preliminary *in vitro* studies were conducted to evaluate the inhibitory activity of the tested inducers on the growth of pathogenic fungi. The results show that *G. catenulatum* was the most efficient biocontrol agent. It reduced the linear growth of *Fusarium solani*, *F. oxysporum*, and *Macrophomina phaseolina* by 76.1, 73.4, and 73.2%, respectively, followed by *B. subtilis* by 71.4, 69, and 68.3%, respectively. Similarly, potassium silicate was the most effective chemical inducer followed by salicylic acid, each at 2000 ppm. They reduced the linear growth of *F. solani* by 62.3 and 55.7%; *M. phaseolina* by 60.7 and 53.1%; and *F. oxysporum* by 60.3 and 53%, respectively. In the greenhouse, all inducers applied as a seed treatment and/or foliar spray strongly limited the development of root rot and wilt diseases. In this regard, *G. catenulatum,* at 1 × 10^9^ CFU mL^−1^, achieved the highest values of disease control, followed by *B. subtilis;* while *T. asperellum,* at 1 × 10^5^ CFU mL^−1^, recorded the lowest values. In addition, the plants treated with potassium silicate followed by salicylic acid, each at 4 g/L, recorded the highest disease control compared to ascorbic acid at 1 g/L, which had the lowest values. The mixture of mycorrhizeen + microbein (at 10 g/kg seeds) was the most effective compared to either of them alone. All treatments, applied alone or in combination in the field, significantly reduced the incidence of diseases. The most effective treatments were a mixture of *G. catenulatum* (Gc) + *Bacillus subtilis* (Bs) + *Trichoderma asperellum* (Ta); a mixture of ascorbic acid (AA) + potassium silicate (PS) + and salicylic (SA); *G. catenulatum*; potassium silicate; and a mixture of mycorrhizeen + microbein. Rhizolix T had the highest disease-reducing efficacy. In response to the treatments, significant improvements in growth and yield, changes in biochemicals, and increased activities of defense enzymes were achieved. This research points to the activity of some biotic and abiotic inducers that can play a vital role in managing the root rot and wilt of roselle through the induction of systemic plant resistance.

## 1. Introduction

Roselle (*Hibiscus sabdariffa* Linn.) is a perennial plant in the Malvaceae family, native to Southern Asia and West Africa, and widespread in the tropics and subtropics [1]. In Egypt it is known as karkade, and in other countries as sorrel, mesta, and Jamaica [2]. The plant is cultivated for its sepals, which are used in the preparation of syrup, jams, and pigments [3]. It has great importance in many culinary, floral, cosmetic, and medical aspects [4]. Egypt, Sudan, Thailand, China, and Mexico are the major producers of roselle blossoms in the world [5]. Roselle is susceptible to many fungal diseases. Among these, root rot and wilt are among the most urgent obstacles to roselle production, as they attack seedlings and mature plants, causing severe losses in yield and quality [6]. The pathogens of such diseases belong to soil-borne fungi [7,8]. To prevent outbreaks of these diseases, many synthetic fungicides and fumigants are used. Although these chemicals are easy to apply, fast-acting, and effective, they pollute the environment and are toxic to humans and animals. In addition, they encourage the development of fungicide-resistant pathogens [9]. Therefore, more balanced, cost-effective, eco-friendly, and farmer-approved approaches need to be applied. One important option is the induction of plant systemic resistance [10].

Plants defend themselves against invading pathogens through a variety of physical and chemical defense mechanisms. This type of resistance enables long-term and broad-spectrum control of fungal, bacterial, and viral diseases using the plants’ natural resistance [11]. Induction of resistance can occur locally in affected tissues or systemically in all plant organs [12]. Plant resistance can be caused by various types of biotic and abiotic inducers [13]. Systemic resistance can be distinguished by two types, the first being systemic acquired resistance (SAR) [14]. This is induced using necrotizing pathogens and synthetic/or natural compounds, such as acibenzolar-S-methyl and hexanoic acid (Hx). It is mediated by a salicylic acid (SA)-dependent process [15]. The second is induced systemic resistance (ISR). This is induced using plant growth-promoting microorganisms and other compounds, such as antibiotics, surfactants, and chemical inducers, such as silica and chitosan [14,16,17]. It is mediated via the jasmonate (JA)- and ethylene (ET)-sensitive pathway [12].

Numerous investigations have shown the potential of beneficial microorganisms, such as *Gliocladium catenulatum* [18,19], *Trichoderma asperellum* [20], and *Bacillus subtilis* [21], to induce systemic plant resistance against phytopathogens. *Gliocladium catenulatum* is known to be a saprophytic filamentous fungus that lives on organic debris. As a rhizosphere-competent and endophyte in the roots and stems of plants, it is highly competitive concerning nutrients and space [22]. It has a high ability to hyperparasitism, destroys mycelium cells, and produces cell wall-degrading enzymes [18,23]. *Trichoderma* species possess a variety of agricultural benefits, such as being a biofertilizer, biofungicide, and bioremediation agent [24]. Their traits include mycoparasitism, competition, and they can induce systemic and localized plant resistance [25]. *Bacillus subtilis*, an endophytic bacterium widespread in nature, can prevent many plant diseases, especially those caused by soil-borne fungi [26]. The biocontrol mechanisms of *B. subtilis* include the synthesis of a variety of antibiotics and hydrolytic enzymes [27], in addition to its high ability to compete for nutrients and space, and stimulate plant systemic resistance [28]. Altogether, microbial biological control agents (MBCAs) protect crops from disease damage through direct mechanisms, such as hyperparasitism and the production of antimicrobial secondary metabolites [23,29], as well as via indirect mechanisms, by inducing systemic resistance or prime immune responses in plant tissues [12,14]. Systemic resistance induced by MBCAs involves multiple physiological and biochemical changes in the host via activating a network of signaling molecules, such as the accumulation of reactive oxygen species, the production of phytoalexins and phenolic compounds, and the formation of physical barriers. In addition, via the stimulation of defense-related enzymes and by influencing the levels of phytohormones, such as jasmine acid (JA) and/or ethylene (ET), which act as key players in the regulation of ISR [14,30,31,32].

Biofertilizers have been used in crop production for decades, as they have a great potential to improve yield through ecofriendly mechanisms [33]. Biofertilizers are products containing microorganisms that, when applied to soil, seeds, or plant surfaces, colonize the rhizosphere or internal tissues of the plant [34]. This is important as this helps in increasing soil fertility, the secretion of growth regulators, the plant’s tolerance to biotic and abiotic stresses, and prevents pathogen attack [35]. Recently, a large number of beneficial microbes have been used in biofertilizer formulation. Among these, plant growth-promoting rhizobacteria (PGPR) and arbuscular mycorrhizal fungi (AMF) [35,36] are more noteworthy. For example, many studies have confirmed that PGPR can suppress phytopathogens by secreting antibiotics, siderophores, and hydrolytic enzymes [28]. They can induce plant resistance in cucumber against Fusarium wilt, angular leaf spot, and root knot nematode [37,38]. In addition, AMF have been shown to provide plants with defensive barriers against soil-borne fungi belonging to *Pythium*, *Phytophthora*, and *Fusarium*, as well as nematodes [39].

Several studies have demonstrated the efficiency of some antioxidants, such as ascorbic acid and salicylic acid, in inducing plant resistance against phytopathogens [40]. These antioxidants can induce several physiological and/or morphological changes in host defense-related compounds, which in turn, increase systemic resistance [41]. For example, treatment with salicylic acid (SA) induced systemic resistance in roselle against root rot and wilt [6], and in tomato against early blight [42]. SA stimulates plant defense responses against pathogens through multiple mechanisms, such as cell wall strengthening, oxidative burst, gene expression regulation, and the induction of pathogenesis-related proteins [11]. In addition, ascorbic acid (AA) was found to be effective in reducing early blight in potato [43], powdery mildew in sunflower [44], and damping-off in tomato [45]. Recently, potassium silicate (K_2_SiO_3_) achieved positive results for inducing the resistance of cotton against Fusarium wilt [46] and cucumber against crown and root rot [47].

The main objective of this study was to evaluate the activity of some biotic and abiotic inducers in suppressing roselle root rot and wilt fungi *in vitro* and *in vivo*, as well as their effect on the growth and yield of roselle, biochemical components, and the activity of defense enzymes. Hence, the tested hypothesis was the possibility of relying on these inducing agents, as alternatives to harmful fungicides, to protect roselle from root rot and wilt fungi.

## 2. Material and Methods

### 2.1. Growing Conditions, Proposed Treatments, and Plant Material

This investigation was designed to evaluate the potential of biotic and abiotic inducers in inducing resistance in roselle against root rot and wilt. The experiments were conducted in the laboratory and farm of the Faculty of Agriculture, Fayoum University, Egypt. The experiment site was located at latitude 29° and 17° N, longitude 30° and 53° E, and 30 m above sea level. Soil samples were brought before the start of the experiment at a depth of 0–30 cm. Soil was subjected to physiochemical analysis according to Olsen and Sommers [48], and the irrigation water to chemical analysis according to Richards [49] (Table 1). The biotic inducers included three biocontrol agents (*Bacillus subtilis*, *Gliocladium catenulatum*, and *Trichoderma asperellum*) and two biofertilizers, i.e., microbein (a mixture of *Pseudomonas*, *Azotobacter*, *Bacillus* and *Rhizobium*) and mycorrhizeen (a mixture of the mycorrhizal spores of *Glomuss*, *Gigaspora*, and *Acaulospora*). The abiotic inducers included ascorbic acid (C_6_H_8_O_6_; molecular weight: 176.124 g mol^−1^), potassium silicate (K_2_SiO_3_; mol. weight: 154.28 g mol^−1^), and salicylic acid (C_7_H_6_O_3_; mol. weight: 138.122 g mol^−1^). The fungicide Rhizolix T 50% WP (chemical name: Tolclofos methyl + thiram) was used for comparison. Roselle seeds (cv. Sobhiya-17) were brought from the Horticultural Research Institute, ARC, Egypt. The dates for sowing seeds in the greenhouse were set during the period from 15 to 20 April 2018 and in the field during the 2019 and 2020 seasons. All roselle farming practices were carried out in accordance with the Ministry of Agriculture and Land Reclamation, Egypt.

### 2.2. In Vitro Studies

#### 2.2.1. Isolation and Identification of Fungi Related to Rotted and Wilted Roselle Samples

Diseased samples were brought from three governorates, namely Fayoum, Beni-Suef, and Minya, and then prepared to isolate the fungal pathogens, as described by Sahi and Khalid [50]. Plant parts were washed, cut, and disinfected with sodium hypochlorite (2%). Small pieces were placed on a sterile PDA supplemented with 0.2% streptomycin, and the plates were then placed in an incubator (25 ± 2 °C) for 3–7 days. Grown cultures were purified using the technique of single spore isolation [51]. Hence, it was identified as described by Barnett and Hunter [52]. Re-isolation from artificially infected plants was performed. Growing colonies were sub-cultured on fresh medium, and identification with original isolates was then confirmed to investigate Koch’s postulations. The frequency was assessed as the percentage of isolated colonies relative to the total isolates.

#### 2.2.2. Source and Inoculum Preparation of Biocontrol Agent Isolates

Isolates of *G. catenulatum*, *T. asperellum*, and *B. subtilis* were provided from the Plant Pathology Research Institute, ARC, Egypt. Fungal isolates were grown on PDA for 7–10 days, then the cultures were immersed in 20 mL of sterile distilled water containing 0.02% Tween-80, and the colony was gently scraped. The suspension was shaken, filtered, and adjusted to 1 × 10^5^, 1 × 10^7^, and 1 × 10^9^ CFU mL^−1^. *Bacillus subtilis* was cultured in liquid nutrient broth in a 250 mL flask and then shaken at 150 rpm for 3 days. Bacterial cells were suspended in sterile water and concentrated to 1 × 10^5^, 1 × 10^7^, and 1 × 10^9^ CFU mL^−1^.

#### 2.2.3. Bioassay of Biocontrol Isolates against Linear Growth of Pathogenic Fungi

The antagonistic activity of the biocontrol isolates on the growth of pathogenic fungi was measured *in vitro* using the dual culture technique described by Dennis and Webster [53]. One agar disc (5 mm-wide) taken from a 7-day-old culture of the biocontrol fungus and transferred into one side of a Petri dish containing sterile PDA. A similar disc, taken from the outer margin of the pathogen, was transferred to the other edge of the plate. The biocontrol bacteria were streaked on one side of the nutrient agar dishes, then placed in an incubator (30 °C) for 48 h. An equal disk (5 mm) of pathogen culture (7 days old) was transferred to the plate in the center. Plates containing pathogens without biocontrol agents were considered as a negative control, as well as those supplemented with Rhizolix T (0.2 g/100 mL PDA) as a positive control. Each treatment was carried out on 5 plates. The reduction in linear growth after 7 days of incubation was calculated using the following equation:

Reduction in linear growth %=[(r1−r2/r1)×100]

where, r1 = growth diameter of the pathogen (GDP) in control and r2 = GDP in treatment.

### 2.3. Pathogenicity Assay, Fungal Inoculum, and Soil Artificial Inoculation

To prepare the pathogenic inoculum, 5–7 discs (5 mm in diameter) of fungal culture (7 days old) were added to a sterile medium of sand and barley individually in large bottles, then placed in an incubator (28 ± 2 °C) for 10 days. To prepare the inoculated soil, sandy loamy soil was sterilized with formalin (5%), covered for a week with polyethylene, and left to dry for 15 days to get rid of traces of formaldehyde. The inoculum was added to the soil at 3% in sterile plastic pots with a diameter of 30 cm. The pots were watered three times, then sown with disinfected roselle seeds (in 1% sodium hypochlorite) at 7 seeds/pot. The control pots contained pathogen-free soil. Diseases were assessed 30-, 60-, and 90-days post-sowing and the surviving plants were counted.

### 2.4. Evaluation of the Inhibitory Activity of Abiotic Inducers on the Linear Growth of Pathogenic Fungi

The activity of the chemical inducers against the linear growth of fungal pathogens was assessed *in vitro* using the technique of food poisoning provided by Kumar et al. [54]. Weights of 0.05, 0.1, and 0.2 g of chemical inducers were mixed individually with 100 mL of PDA before solidification in conical flasks, then shaken to obtain 500, 1000, 2000 ppm, respectively. Streptomycin 0.2% was added to the medium, which was poured into Petri dishes and left to solidify. One agar disc (5 mm in diameter) of pathogen culture (7 days old) was transferred to the plate in the center. Plates containing pathogens without chemicals were kept as a negative control, as well as those treated with Rhizolix T as a positive control. Each treatment was carried out on 5 plates. The reduction in linear growth was calculated using the following equation:

Reduction in linear growth %=[(c1−c2/c1)×100]

where, c1 = colony diameter (CD) in the control and c2 = CD in the treatment.

### 2.5. Induction of Systemic Resistance in Roselle against Root Rot and Wilt Diseases under In Vivo Conditions

Pot and field experiments were planned to estimate the activity of the biotic and abiotic inducers against the root rot and wilt of roselle. In the pot experiment, the seeds were disinfected, immersed in a 5% Arabic gum solution, and individually soaked for 8 h in 1, 2, and 4 g/L of chemical inducer solutions, and in 1 × 10^5^, 1 × 10^7^, and 1 × 10^9^ CFU mL^−1^ of biocontrol agent suspensions. Microbein, mycorrhizeen, and their mixture at 10 g/kg seeds, as well as Rhizolix T (3 g/kg seeds) were used as a seed coating only. After treatment, the seeds were sown in inoculated soil in pots (7 seeds/pot). Foliar spraying with the tested inducers (at the same rates used for soaking the seeds) was performed three times at one-week intervals, the first starting at the growth stage of 2–4 true leaves. A control was kept without treatment. The experiment was completely randomized (3 replicates/treatment, 5 pots/replicate). In the field experiment, the treatments were applied alone or in combination as seed treatment and/or foliar spray at the highest rates used in the greenhouse. The experiment was performed in randomized complete blocks (3 replicates/treatment). The plot size was 12 m^2^ (3 × 4 m) with four rows; spaced 50 cm apart. The seeds were sown in hills (4 seeds/hill) 30 cm apart.

### 2.6. Root Rot/Wilt Disease Measurements

The growing roselle was monitored in the greenhouse or in the field at the pre- and post-emergence stages, 15 and 28 days after sowing, respectively, to estimate the incidence of damping-off using the following equations:

Pre emergence damping−off %=[(No. of non emerged seedlings/No. of seedss own)×100]

Post emergence damping−off %=[(No. of infected seedlings/Total no. of seedlings)×100]

Disease incidence and disease control were estimated periodically every 10 days, starting at 60 to 90 days post-sowing using the following formulas:

Disease incidence (DI) %=[(No. of diseased plants/Total no. of plants)×100]

Disease control %=[(DI in control−DI in treatment/DI in control)×100]

The surviving plants were assessed using the following equation:

Surviving plants %=[(No. of surviving plants/Total no. of diseased plants)×100]

### 2.7. Monitoring the Quality of Growth and Yield

At the full flowering stage (160 days post-sowing), roselle plant height (cm) and the number of branches were measured. The number of fruits/plot, fresh and dry weight of sepals (kg/plot), and the dry weight of seeds (kg/plot) were detailed at the harvesting stage (210 days post-sowing). Plant materials were dried in an electric oven (70 ± 2 °C) for 48 h. The total yield was calculated by counting the fruits, weighing the sepals and seeds, and converting the yield for each plot.

### 2.8. Estimation of Biochemical Changes

#### 2.8.1. Defensive Enzyme Activity Assay

Pre-weighed dry tissues of leaves were homogenized in 4 mL buffer (50 mmol L^−1^ Tris pH ¼ 8.5 and 14.4 mmol L^−1^ 2-mercaptoethanol) and 1% insoluble polyvinylpolypyrrolidone. The mixture was centrifuged at 6000× *g* for 15 min, and then the total protein of the extract was measured using the method described by Bradford [55].

##### Peroxidase (POX)

POX activity was assessed using the method of Dazy et al. [56]. The intensity of the photometric reaction was estimated using a spectrophotometer (470 nm) in a 40 mmol L^−1^ H_2_O_2_ solution. The results were recorded as the POX U mg protein^−1^ min^−1^.

##### Polyphenol Oxidase (PPO)

PPO activity was measured using the method described by Constabel and Ryan [57]. Supernatant was added to the substrate (5 mg mL^−1^ L-3,4-dihydroxyphenylalanine). The assay solution was composed of 100 mmol L^−1^ NaPO_4_ (pH 7), 0.015% NaC_12_H_25_SO_4_, and catalase (280 U mL^−1^). Absorbance was read at 490 nm and the results were recorded as the PPO U mg protein^−1^ min^−1^.

##### Phenylalanine Ammonia-Lyase (PAL)

PAL activity was assessed using the method described by Beaudoin-Eagan and Thorpe [58]. The intensity of the photometric reaction was estimated using a spectrophotometer (290 nm) with 15 mmol L^−1^ phenylalanine (as the substrate). The results were recorded as the PAL U mg protein^−1^ min^−1^.

#### 2.8.2. Phytochemical Parameters

##### Total Soluble Carbohydrates (TSC)

The TSC was determined in the dry leaves according to the method described by Dubois et al. [59]. The sample (100 mg) was mixed with 80% ethanol (3 mL). The mixture was kept at room temperature for 48 h, and then the ethanol was evaporated. Distilled water (20 mL) was added to the dry residue, then 4 mL of anthrone reagent was added to 2 mL of extract and placed in a water bath at 62 °C for 8 min. The tube was placed to rest in the dark for 30 min, and the absorbance was read at 490 nm.

##### Total Anthocyanin Concentration (TAC)

TAC was assessed using the method of Chew et al. [60]. In total, 1 mL of 0.2 M/L KCl_2_ solution (pre-adjusted to pH 1.0) or 1 M/L sodium acetate buffer (pre-adjusted to pH 4.5) was added to 2 mL of extract, respectively. Absorbances were read at 517 and 700 nm. The results were recorded as mg C3G equivalent/100 g extract. Anthocyanin was calculated using the next equation:

Anthocyanin concentration (mg/L) = [A × E × L × MW × 10^3^ × D]

where, A = difference between the absorbance of the sample at pH 1.0 and 4.5, E = molar extinction coefficient for cyanidin-3-glucoside (26,900), L = path length (1 cm), MW = molecular weight of C3G (449.2 g/mol), 10^3^ = conversion from g to mg, and D = dilution factor.

##### Vitamin C

Vitamin C was assessed according to Wimalasir and Wills [61]. The sample (4 mL) was mixed with methanol (4 mL) and distilled water (10 mL), and then the mixture was filtered with a 0.45 µm filter. After the first 3 mL of the filter, 1 mL was collected for analysis. The sample (20 mL) was injected into the HPLC system. The effluent was monitored in the column at 254 nm. The results were recorded as mg vitamin c 100 g^−1^ DW.

##### Total Acidity %

The total acidity (titratable acidity) was assayed by Gbadegesin et al. [62]. The sample (1 g) was added to distilled water (40 mL), then the mixture was heated to 70 °C and left to stand for 1 h. The supernatant was filtered and the roselle residue was rinsed with hot distilled water, then the filtrate was cooled. A 25 mL aliquot of the extract was titrated with 0.1 N NaOH to a pH of 8.3. The results were recorded as a malic acid% as in the next equation:

$$Total\;acidity\left(malic\;acid\%\right)=\left(\frac{Volume\;of\;titration\times 0.1\;N\;NaOH\times 0.067\times 100}{Volume\;of\;sample}\right)$$

### 2.9. Statistical Analysis of the Data

All data were analyzed statistically with ANOVA, using the Web Agricultural Statistics Software Package (WASP 2.0, ICAR Research Complex, Goa, India). The values shown are the means of all proposed measurements. A combined analysis of the data collected during the two growing seasons and Duncan’s range test were used to compare significant differences between all treatments at *p* ≤ 0.05.

## 3. Results

### 3.1. Isolation and Identification of Fungi Related to Roselle Root Rot and Wilt Diseases

As shown in Table 2, five fungi belonging to three fungal genera were isolated from roselle infected with root rot and wilt, obtained from three locations in Egypt during the 2017 season. The fungal isolates were purified and identified as the following fungi: *Fusarium equiseti* (Corda) Sacc., *F. oxysporum* (Schlecht.) Snyder and Hansen, *F. solani* (Mart.) Sacc., *Macrophomina phaseolina* (Tassi) Goid., and *Pythium ultimum* Trow. Roselle artificially diseased with isolated fungi showed the same characteristic symptoms of root rot and wilt observed previously. Re-isolation trials led to obtaining the same original isolates. Our findings also showed that *F. oxysporum* had the most frequency followed by *F. solani*, *M. phaseolina*, and *F. equiseti*. The corresponding frequency values were 38.9, 24.1, 22.2, and 10.2%, respectively, while *P. ultimum* recorded the lowest value of 4.6%.

### 3.2. Pathogenicity Assay of Root Rot and Wilt-Related Fungi against Roselle Seedlings under Greenhouse Conditions

The data in Table 3 show that all tested fungi were pathogenic to roselle seedlings, causing damping-off, root rot and wilt. Infection increased with increasing plant age from 30 to 90 days after sowing. In this regard, *M. phaseolina* had the most pathogenicity, followed by *F. oxysporum* and *F. solani*. The corresponding values of the surviving plants were 37.5, 41.7, and 45.8%, respectively, while *P. ultimum* and *F. equiseti* recorded the lowest values of 70.8 and 66.7%, respectively.

### 3.3. In Vitro, Inhibitory Activity of Biotic Inducers against the Linear Growth of Roselle Root Rot and Wilt Fungi

All of the biocontrol agents showed potent inhibitory activity against the growth of fungal pathogens (Table 4). The highest reduction in linear growth was recorded using *G. catenulatum* followed by *B. subtilis*. They reduced the linear growth of *F. solani* by 76.1 and 71.4%; *F. oxysporum* by 73.4 and 69%; and *M. phaseolina* by 73.2 and 68.3%, respectively, while *T. asperellum* recorded the lowest values of 62.5, 64.5, and 64.7% for *M. phaseolina*, *F. solani*, and *F. oxysporum*, respectively. The fungicide Rhizolix T (positive control) outperformed all treatments, reducing the growth of *F. solani*, *F. oxysporum*, and *M. phaseolina* by 89.8, 89.4, and 87.2%, respectively.

### 3.4. In Vitro, Inhibitory Activity of Abiotic Inducers against the Linear Growth of Roselle Root Rot and Wilt Fungi

All of the chemical inducers significantly reduced the linear growth of the fungal pathogens (Table 5). Potassium silicate recorded the highest reduction followed by salicylic acid, each at 2000 ppm. The corresponding values were 62.3 and 55.7% for *F. solani*; 60.7 and 53.1% for *M. phaseolina*; and 60.3 and 53% for *F. oxysporum*, respectively, while ascorbic acid at 500 ppm recorded the lowest reduction of 14.3, 16.4, and 20.7% for *F. oxysporum*, *M. phaseolina*, and *F. solani*, respectively. Treatment with Rhizolix T at 2000 ppm showed remarkable superiority over all treatments, reducing the linear growth of *F. oxysporum*, *M. phaseolina*, and *F. solani* by 89.8, 88.3, and 88.1%, respectively.

### 3.5. Potential of Biocontrol Agents, Chemical Inducers, and Biofertilizers against Root Rot and Wilt of Roselle under Greenhouse Conditions

The data in Table 6 indicated that all of the treatments significantly reduced the incidence of root rot and wilt of roselle. In this regard, *G. catenulatum* was the most efficient biocontrol agent followed by *B. subtilis*, each at 1 × 10^9^ CFU mL^−1^. They reduced the damping-off caused by *F. solani* by 78 and 70.1%, *F. oxysporum* by 75.3 and 70%, and *M. phaseolina* by 72.3 and 65%, respectively, and root rot/wilt caused by *F. oxysporum* by 78.2 and 75.2%, *M. phaseolina* by 75.7 and 70%, and *F. solani* by 73.7 and 68.2%, respectively. *T. asperellum,* at 1 × 10^5^ CFU mL^−1^, recorded the lowest values. In addition, plants treated with potassium silicate and salicylic acid, each at 4 g/L, recorded the highest disease control. They reduced the damping-off caused by *F. solani* by 72.1 and 61.3%, *M. phaseolina* by 68 and 60.8%, and *F. oxysporum* by 64 and 57.6%, respectively, and root rot/wilt caused by *F. oxysporum* by 75 and 69%, *M. phaseolina* by 73.7 and 65.9%, and *F. solani* by 65 and 63.5, respectively. Ascorbic acid, at 1 g/L, recorded the lowest values. In a similar vein, the mixture of mycorrhizeen + microbein was the most effective biofertilizer followed by mycorrhizeen, each at 10 g/kg seeds. They reduced the damping-off caused by *M. phaseolina* by 59.2 and 54.5%, *F. oxysporum* by 55.4 and 49.2%, and *F. solani* by 52.5 and 44.1%, respectively, and root rot/wilt caused by *M. phaseolina* by 68 and 61.3%, *F. solani* by 64.4 and 56.3%, and *F. oxysporum* by 59.2 and 53.4%, respectively, while microbein recorded the lowest values. Rhizolix T fungicide showed remarkable superiority in disease reduction compared to all treatments.

### 3.6. Induction of Systemic Resistance in Roselle against Root Rot and Wilt under Field Conditions

The data in Table 7 shows that all treatments, used alone or in combination, significantly reduced the incidence of root rot and wilt diseases. The most effective treatments used were the mixture of Gc + Bs + Ta; the mixture of AA + PS + SA; *G. catenulatum*; potassium silicate; and the mixture of mycorrhizeen + microbein. They reduced the pre-emergence damping-off by 79.2, 77, 73.2, 67.2, and 59.6%, respectively; post-emergence damping-off by 72.5, 68, 60.4, 56.4, and 50.1%, respectively; root rot by 75.6, 70.4, 64.2, 59.9, and 53.4%, respectively; and wilt by 77.2, 73.2, 70.4, 66, and 59.5%, respectively. Ascorbic acid recorded the lowest disease control for pre-emergence damping-off (39.1%), post-emergence damping-off (30%), root rot (28.3%), and wilt (32.5%). The fungicide Rhizolix T had the highest disease-reducing efficacy than all treatments.

### 3.7. Quality of Growth and Yield of Treated Roselle

**Figure 1:**
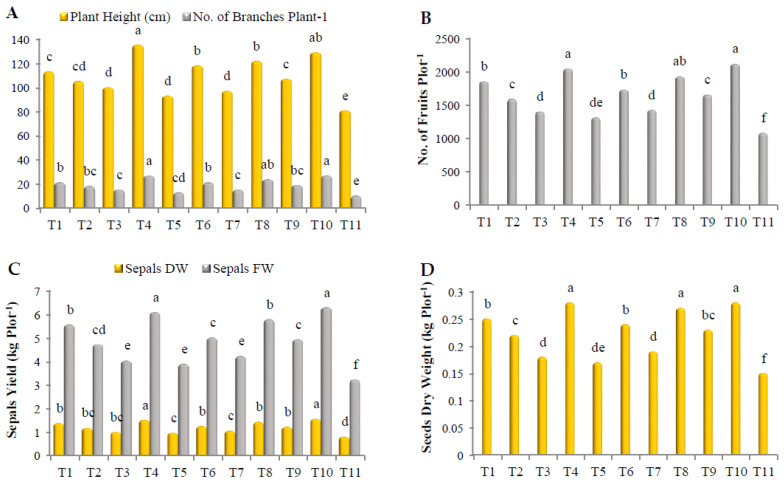
Effect of treatments on (**A**) plant height and the number of branches per plant^−1^; (**B**) number of fruits per plot^−1^; (**C**) fresh and dry weight of sepals per plot^−1^; and (**D**) dry weight of seeds per plot^−1^ of roselle. The data collected are the mean of two repeated experiments during the 2019 and 2020 seasons. Different letters on the columns show significant differences between the treatments as per Duncan’s multiple ranges test at *p* ≤ 0.05 statistical level.

As shown in Figure 1, all treatments improved roselle growth and yield. Plants treated with a mixture of Gc + Bs + Ta, Rhizolix T, and a mixture of AA + PS + SA showed a remarkable superiority in plant height, with significant differences, recording 135.3, 129.2, and 122.1 cm, respectively. The plants treated with ascorbic acid (AA) and salicylic acid (SA) were found in the lowest order (Figure 1A). In addition, treatment with Rhizolix T and the mixture of Gc + Bs + Ta recorded the highest number of branches/plant, without significant differences, recording 27.1 and 27, respectively, followed by the mixture of AA + PS + SA, *G. catenulatum*, and potassium silicate, with significant differences, recording 24, 21.3, and 21.3, respectively. The least value was noted using AA (13.1) (Figure 1A). In a similar vein, all treatments significantly increased the number of fruits/plot, specifically Rhizolix T, the mixture of Gc + Bs + Ta, the mixture of AA + PS + SA, *G. catenulatum*, potassium silicate, and the mixture of mycorrhizeen + microbein, with significant differences. The corresponding values were 2120, 2047.2, 1932.1, 1858.2, 1733.1, and 1654.1, respectively. AA was the lowest (1315.1) (Figure 1B). Regarding the fresh and dry weight of sepals/plot, the best results were recorded with Rhizolix T, the mixture of Gc + Bs + Ta, the mixture of AA + PS + SA, *G. catenulatum*, and potassium silicate. They recorded 6.31, 6.10, 5.79, 5.57, and 5.02 kg, respectively, in the fresh weight of sepals, and 1.57, 152, 144, 139, and 126 kg, respectively, in the dry weight of sepals (Figure 1C). The highest dry weight of seeds/plot was recorded using Rhizolix T, the mixture of Gc + Bs + Ta, and the mixture of AA + PS + SA, without significant differences, recording 0.28, 0.28, and 0.27 kg, respectively, followed by *G. catenulatum*, potassium silicate, the mixture of mycorrhizeen + microbein, and *B. subtilis*, recording 0.25, 0.24, 0.23, and 0.22 kg, respectively. AA was the lowest (0.17 kg) (Figure 1D).

### 3.8. Defensive Enzyme Activities

**Figure 2:**
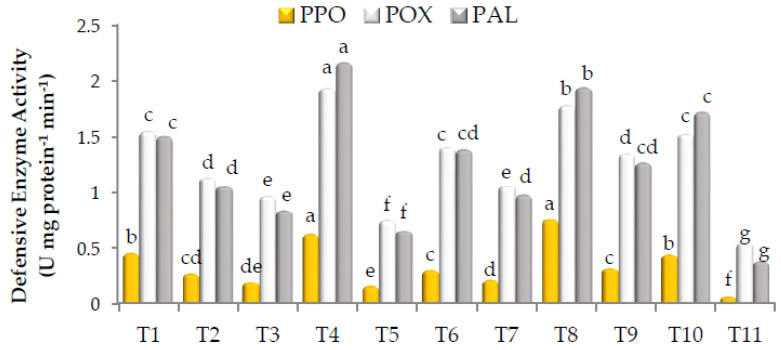
Activity of peroxidase (POX), polyphenol oxidase (PPO), and phenylalanine ammonia-lyase (PAL) in roselle. The data collected are the mean of two repeated experiments during the 2019 and 2020 seasons. Different letters on the columns show the significant differences between the treatments as per Duncan’s multiple ranges test at *p* ≤ 0.05 statistical level.

As shown in Figure 2, all treatments significantly increased the activity of POX, PPO, and PAL. The highest activity of POX was estimated in the plants treated with the mixture of Gc + Bs + Ta, the mixture of AA + PS + SA, *G. catenulatum*, Rhizolix T, potassium silicate, and the mixture of mycorrhizeen + microbein, with significant differences. The corresponding values were 1.935, 1.781, 1.549, 1.524, 1.405 and 1.347 Unit mg protein^−1^ min^−1^, respectively. Similarly, the highest PPO activity was found in the plants treated with the mixture of AA + PS + SA, the mixture of Gc + Bs + Ta, and *G. catenulatum*, with significant differences, recording 0.755, 0.623 and 0.451 Unit mg protein^−1^ min^−1^, respectively. In addition, the highest PAL activity was recorded with the mixture of Gc + Bs + Ta, the mixture of AA + PS + SA, Rhizolix T, and *G. catenulatum*, with significant differences. The corresponding values were 2.167, 1.942, 1.721 and 1.503 Unit mg protein^−1^ min^−1^, respectively. However, the lowest POX, PPO and PAL activities of 0.748, 0.156 and 0.652 Unit mg protein^−1^ min^−1^, respectively, were recorded with ascorbic acid.

### 3.9. Induction of Biochemical Changes in Roselle Plant

**Figure 3:**
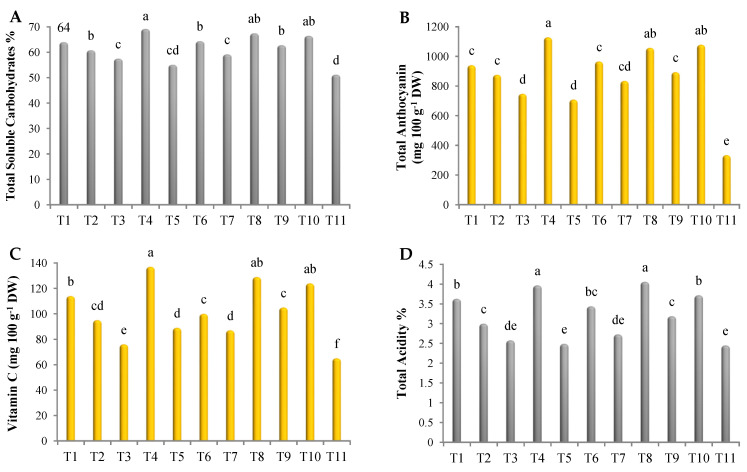
Content of (**A**) total soluble carbohydrates; (**B**) total anthocyanin; (**C**) vitamin C; and (**D**) total acidity in roselle. The data collected are the mean of two repeated experiments during the 2019 and 2020 seasons. Different letters on the columns show the significant differences between the treatments as per Duncan’s multiple ranges test at *p* ≤ 0.05 statistical level.

As shown in Figure 3, all treatments induced changes in the biochemical components of roselle. In this regard, the highest amounts of total soluble carbohydrates were noted in plants treated with the mixture of Gc + Bs + Ta, the mixture of AA + PS + SA, Rhizolix T, *G. catenulatum*, and potassium silicate, without significant differences, recording 69.2, 67.5, 66.5, 64, and 64%, respectively (Figure 3A). Regarding total anthocyanin, treatment with the mixture of Gc + Bs + Ta, Rhizolix T, the mixture of AA + PS + SA, potassium silicate, and *G. catenulatum* recorded the highest values, with significant differences, recording 1129, 1079, 1058, 966, and 941 mg 100 g^−1^ DW, respectively (Figure 3B). In addition, the highest amounts of vitamin C were found in plants treated with the mixture of Gc + Bs + Ta and the mixture of AA + PS + SA, with significant differences, followed by Rhizolix T, *G. catenulatum*, and the mixture of mycorrhizeen + microbein. The values were 137, 129, 124, 114, and 105 mg 100 g^−1^ DW, respectively (Figure 3C). Similarly, the highest values of total acidity were recorded using the mixture of AA + PS + SA and the mixture of Gc + Bs + Ta, without significant differences, recording 4.06 and 3.97%, respectively, followed by Rhizolix T (3.72%), *G. catenulatum* (3.63%), potassium silicate (3.44%), and the mixture of mycorrhizeen + microbein (3.19%) (Figure 3D). However, treatment with ascorbic acid, *T. asperellum*, and salicylic acid recorded the lowest values.

## 4. Discussion

Soil-borne root rot and wilt are some of the most severe diseases affecting many crops worldwide, resulting in poor production and quality, and low agricultural income [63]. In Egypt, such diseases are among the most urgent obstacles to roselle production, as they attack seedlings and mature plants, causing severe yield losses [6]. Our results showed that among five fungi related to roselle root rot and wilt, *F. oxysporum* was the most frequent followed by *F. solani*, *M. phaseolina*, and *F. equiseti*. In addition, *M. phaseolina* was the most pathogenic fungus followed by *F. oxysporum* and *F. solani*. These results have been previously supported by [7,8]. The management of root rot or wilt diseases is an ongoing challenge for growers, because pathogens survive in or near the rhizosphere for a long time in the soil by forming resistant structures. Moreover, the physical, biological, and structural complexity of the soil’s micro-ecosystem limits the options for controlling these diseases [64]. To date, the principal means of controlling these diseases remains the use of fungicides. However, this strategy is no longer appropriate due to health and environmental risks, in addition to the development of fungicide-resistant fungi [9]. The induction of plant resistance is one of the agricultural strategies with the most potential for preventing biotic losses. It enables the long-term and broad-spectrum control of bacterial, fungal, and viral diseases using the plant’s natural resistance [11]. This resistance can be caused by various types of biotic and abiotic inducers [13]. Our *in vitro* studies showed that *B. subtilis*, *G. catenulatum*, and *T. asperellum* exhibited potent linear growth inhibitory activity of fungal pathogens. The highest reduction was recorded using *G. catenulatum* followed by *B. subtilis*. These results were similar to those of Tut et al. [19], who found that *G. catenulatum* reduced *B. cinerea* mycelial growth. In addition, *B. subtilis* has been found to be effective in reducing mycelium growth and the germination of spores of many pathogenic fungi, including *B. cinerea*, *F. oxysporum*, *F. solani*, *M. phaseolina*, *Alternaria solani*, and *Phytophthora infestans* [6,19,65]. According to Yi et al. [21], *B. subtilis* filtrates significantly inhibited the mycelial growth of *Rhizoctonia cerealis*, causing swelling and rupture of the mycelium, disturbing the permeability of the cell membranes, and thus, destroying organelles. Along the same line, *T. asperellum* showed high mycelial growth inhibitory activity against *F. oxysporum,* up to 77.4% [20]. Our results also showed that biocontrol agents significantly decreased the incidence of root rot and wilt of roselle *in vivo*. This finding is similar to that of Hassan et al. [6], who found that soaking roselle seeds in *B. subtilis* and *T. harzianum* suspensions induced plant resistance against root rot and wilt infection. Similarly, McQuilken et al. [18] mentioned that treating bedding seeds with *G. catenulatum* stimulated systemic resistance against damping-off. The potential of *G. catenulatum* as a biocontrol agent can be attributed to the intense competition for nutrients and space, hyperparasitism, destruction of mycelium cells, and secretion of cell wall-degrading enzymes [18,22,23]. According to Sun et al. [66], *G. catenulatum* hyphae can grow along and around the hyphae of *Pythium ultimum* and *R. solani*, penetrating into them and then destroying their cells by producing perilipin protein. As for *B. subtilis*, it has the ability to produce a broad spectrum of antibiotics (i.e., lipopeptides and cyclic peptides) and hydrolytic enzymes (i.e., glucanase, protease, and chitinase), enabling it to degrade the cell walls of the mycelium and inhibit the germination of spores [27]. *Bacillus subtilis* also competes vigorously for nutrients and space and induces a plant’s systemic resistance [28]. In a similar vein, *Trichoderma* spp. is considered a potent biocontrol agent due to its mycoparasitism, competition, and ability to induce systemic and localized plant resistance [25]. Moreover, it produces a number of antibiotics and cell wall-degrading enzymes [67]. These substances intensively inhibit the germination of spores and the elongation of germ tubes [68].

Our *in vitro* studies indicated that ascorbic acid, potassium silicate, and salicylic acid significantly inhibited the linear growth of fungal pathogens. The highest reduction was recorded using potassium silicate followed by salicylic acid. A number of studies showed the effectiveness of potassium silicate in inhibiting the mycelium growth of *R. solani*, *F. solani*, *F. oxysporum*, *F. equiseti*, and *F. semitectum* [69], and the germination of powdery mildew conidia by 40–60% [70]. In addition, the application of salicylic and ascorbic acids caused a significant decrease in the linear growth of *F. oxysporum*, *F. solani*, and *M. phaseolina* [6]. Similarly, the combination of salicylic acid, chitosan, and humic acid reduced the spore germination and mycelium growth of *R. solani* and *F. solani* [63]. Our results also showed that all chemical inducers significantly reduced the incidence of root rot and wilt of roselle *in vivo*. Several reports have indicated the efficacy of potassium silicate in inducing a plant’s systemic resistance against fungal diseases. Among these, Whan et al. [46] found that treating cotton with potassium silicate induced resistance against *F. oxysporum* f. sp. *vasinfectum* by increasing phenol content and lignin formation. Likewise, Chérif et al. [47] found that treating cucumber with potassium silicate induced resistance against root rot by increasing the activity of ß-glucosidase and fungi-toxic aglycones. This activity of potassium silicate is due to the combined effect of potassium and silicon. Silicon increases the activities of defense enzymes and antimicrobial compounds, including phytoalexins, phenols, and pathogenicity-related proteins. It regulates host resistance via signaling hormones, i.e., salicylic acid (SA), jasmonic acid (JA), and ethylene (ET) [40]. Potassium improves the health and vigor of the plant, making infection less likely and aiding in rapid recovery [71]. According to Abd-El-Kareem et al. [72], potassium likely exerts its effect on plant disease via some metabolic functions, altering parasite–host environment relationships, and producing pathogen-inhibiting compounds (i.e., phenols, phytoalexins, and auxins). Several studies have confirmed the efficacy of ascorbic acid in reducing early blight in potato [43], powdery mildew in sunflowers [44], and damping-off in tomato [45]. In addition, treatment with salicylic acid (SA) induced systemic resistance in roselle against root rot and wilt [6] and in tomato against early blight [42]. SA stimulates the plant’s defense responses against pathogens through multiple mechanisms, such as cell wall strengthening, oxidative burst, gene expression regulation, and the induction of pathogenesis-related proteins [11]. Moreover, it regulates ethylene, jasmine acid, and auxin signaling [73]. SA promotes the accumulation of phytoalexins (toxic to pathogens) [74]. Our investigation showed that the mixture of mycorrhizeen + microbein was the most effective biofertilizer followed by mycorrhizeen. Biofertilizer microbes can colonize roots and protect plants indirectly by stimulating plant growth through improving soil fertility and structure, facilitating nutrient uptake, tolerating abiotic stress, and secreting growth regulators [75], or directly by protecting plants from pathogens. Plant growth rhizobacteria (PGPR) can resist pathogens by secreting antibiotics, siderophores, and hydrolytic enzymes [29]. These substances break down the hyphae and spores of pathogens [76]. In addition, a number of studies have indicated the potential of arbuscular mycorrhizal fungi (AMF) to suppress soil-borne fungi, such as *Pythium*, *Phytophthora*, and *Fusarium* [39]. AMF can colonize roots and form a fungal mat that provides a physical barrier against pathogens, compete with pathogens, and secrete antagonistic chemicals [77]. The present results showed the superiority of the Rhizolix T fungicide in disease control compared to all treatments. This activity is likely due to the fungicide interfering with cell wall biosynthesis, increasing cell wall permeability, destroying the plasma membrane, and preventing the biosynthesis of ergosterol, which is essential for cell wall synthesis, thus damaging the cell wall [78].

Our results indicated that all biotic and abiotic inducers improved the growth and yield of roselle. These findings are consistent with a number of previous studies [6,79]. In terms of biotic inducers, bacteria belonging to the PGPR group improve the growth of the plant via multiple mechanisms, including biofilm formation [80], synthesis of siderophores and phytohormones [29], fixation of nitrogen [81], production of vitamins, amino acids, and antioxidants [82], synthesis of ACC-deaminase (ACCD) that reduces ethylene production in roots [83], and the production of plant growth regulators, i.e., gibberellic acid, cytokinins, and indole-3-acetic acid [75]. In addition, PGPF, such as arbuscular mycorrhizal fungi, produce hormones that allow a plant–soil interaction, decomposing organic matter through the solubilization of minerals [84]. With respect to abiotic inducers, salicylic acid (SA) stimulates plant growth by regulating several physiological and biological functions, i.e., seed germination, flower growth regulation, fruit maturing, sex differentiation, stomata movement, and photoperiod [85]. SA modulates cell membrane permeability, stomatal conduction, ion uptake, growth progression, and transport [86]. Likewise, ascorbic acid is involved in cell division and expansion, photoprotection, photosynthesis, and flowering [87]. It also contributes to metabolic and cell signaling activities as well as regulating many physiological functions that control tolerance to various stresses [88]. In addition, potassium silicate is used as a source of potassium (K) and soluble silicon (Si) and as a plant stimulant. Silicon improves root structure, leaf erection, photosynthesis, and water relations [40]. Potassium performs many functions, such as protein and starch synthesis, cell division, seed size and quality. Moreover, it stimulates root length and vegetative growth, regulates osmosis, and enhances chlorophyll pigments, stomata movement and water status [71,89]. In this study, we found that all treatments significantly increased the activity of the POX, PPO, and PAL enzymes. Our finding is similar to that of Chowdappa et al. [65], who found that the use of *B. subtilis* and *T. harzianum* resulted in tomato resistance to early and late blight by enhancing the activity of the PPO, POX, and SOD enzymes. Similarly, the treatment of sunflower with a mixture of ascorbic acid, salicylic acid, *T. harzianum*, and *B. subtilis* triggered ISR against powdery mildew by increasing the POX, PPO, and CAT enzymes [44]. POX plays a vital role against pathogens via multiple mechanisms, i.e., regulation of the synthesis and accumulation of antimicrobial substances, plant cell elongation, oxidation of phenols and IAA, cross-linking of polysaccharides, and the oxidation of hydroxyl-cinnamyl alcohol to free radical intermediates [90]. Moreover, it stimulates the formation of lignin, which provides rigidity and strength to the plant cell wall against biological, chemical, or physical attacks [91]. As for PPO, it oxidizes phenols to antimicrobial quinines (more toxic than phenols) [92]. It also promotes the deposition of lignin onto plant cell walls, contributing to the formation of defensive barriers against pathogen attack [93]. In addition, PAL contributes to the biosynthesis of SA, phytoalexin, phenols, and lignin [94]. Our results also showed that all of the inducers prompted changes in the biochemical components of roselle, including total soluble carbohydrates, total anthocyanin, vitamin C, and total acidity. These findings are consistent with those of Al-Sayed et al. [95], who found that the treatment of roselle seeds with *Azorobacter* and *Azospirillum* significantly increased the contents of anthocyanin, chlorophyll, carotenoid, and flavones. Similarly, Abo-Baker and Mostafa [96] revealed that the application of *B. polymyxa* and *Azospirillum* increased anthocyanin, vitamin C, and the total acidity in roselle. In a similar vein, Selem et al. [97] found that spraying potato with ascorbic and salicylic acids significantly increased the plant-soluble sugars and total carbohydrates in the tubers.

## 5. Conclusions

Our study declared that all biotic and abiotic inducers were used effectively to induce systemic resistance in roselle against root rot and wilt. All treatments showed potent inhibitory activity against the linear growth of pathogenic fungi *in vitro*, and strongly limited disease development when applied as seed treatments and/or via foliar spraying in the greenhouse. In addition, treatments applied alone or in combination in the field significantly reduced the incidence of diseases. The most effective treatments were the mixture of Gc + Bs + Ta, the mixture of AA + PS + SA, *G. catenulatum*, potassium silicate, and the mixture of mycorrhizeen + microbein. They led to significant improvements in growth and yield, changes in biochemicals, and the increased activities of defense enzymes. In conclusion, the induction of systemic host resistance has become a good target for suppressing the root rot and wilt of roselle and promoting crop growth without environmental risk.

## Figures and Tables

**Table 1:** Soil physiochemical analysis and irrigation water chemical analysis during two experimental growing seasons 2019 and 2020.

**Soil Physiochemical Analysis**	**2019 Season**	**2020 Season**
Clay %	12.3	13.54
Silt %	17.48	18.11
Sand %	70.22	68.35
Soil texture	Sandy loam	Sandy loam
pH (1:2.5)	7.5	7.35
EC dS m^−1^	3.67	3.9
Organic matter %	0.98	1.12
CaCO_3_ %	4.92	5.13
Total N %	0.09	0.12
Available P mg kg^−1^ soil	7.68	7.91
Available K mg kg^−1^ soil	179	183
Available Fe mg kg^−1^ soil	6.4	6.35
**Irrigation Water Analysis**		
pH	5.21	4.93
EC dS m^−1^	7.04	6.95
CO_3_^−^ meq/L	0	0
HCO_3_^−^ meq/L	2.17	2.5
SO_4_^−^ meq/L	21.05	20.42
Cl^−^ meq/L	40.36	38.9
Ca^++^ meq/L	5.57	6.14
Mg^++^ meq/L	13.04	14.52
Na^+^ meq/L	44.19	41.31
K^+^ meq/L	0.26	0.29

**Table 2:** Identification and frequency values of fungi isolated from roselle infected with root rot and wilt, obtained from three locations in Egypt during the summer 2017 season.

Isolated Fungi	Location/Number of Isolates and Frequency Values							
	Fayoum Governorate		Beni-Suef Governorate		Minya Governorate		Total	
	No. of Isolates	Frequency%	No. of Isolates	Frequency %	No. of Isolates	Frequency %	No. of Isolates	Frequency %
*F. equiseti*	3	7.5	3	8.6	5	15.2	11	10.2
*F. oxysporum*	14	35.0	17	48.6	11	33.3	42	38.9
*F. solani*	12	30.0	7	20.0	7	21.2	26	24.1
*M. phaseolina*	9	22.5	8	22.8	7	21.2	24	22.2
*P. ultimum*	2	5.0	0	0.0	3	9.1	5	4.6
Total	40	100	35	100	33	100	108	100

Fayoum Governorate: latitude 29° and 17° N, longitude 30° and 53° E, and 30 m above sea level; Beni-Suef Governorate: latitude 29° and 3° N, longitude 31° and 5° E, and 32.4 m above sea level; and Minya Governorate: latitude 28° and 4° N, longitude 30° and 3° E, and 40 m above sea level.

**Table 3:** Pathogenicity assay of isolated fungi against roselle seedlings (cv. Sobhiya-17) after 30, 60, and 90 days of sowing under greenhouse conditions.

Tested Fungi	Disease Infection %			* Surviving Plants %
	Days after Sowing			
	30 Days	60 Days	90 Days	
*F. equiseti*	16.7 ± 0.74 ^d^	25.0 ± 1.0 ^d^	33.3 ± 1.30 ^d^	66.7
*F. oxysporum*	25.0 ± 1.06 ^b^	37.5 ± 1.41 ^b^	58.3 ± 1.0 ^b^	41.7
*F. solani*	20.8 ± 0.66 ^c^	33.3 ± 1.14 ^c^	54.2 ± 1.10 ^c^	45.8
*M. phaseolina*	29.2 ± 0.91 ^a^	41.7 ± 0.92 ^a^	62.5 ± 0.50 ^a^	37.5
*P. ultimum*	20.8± 0.76 ^c^	25.0± 1.02 ^d^	29.2± 1.10 ^e^	70.8
Control (pathogen-free soil)	0.0 ± 0.0 ^e^	0.0 ± 0.0 ^e^	0.0 ± 0.0 ^f^	100

* The values of the surviving plants were assessed based on the total number of diseased plants, 90 days post-sowing. Within the same column, values followed by the same letters are not significantly different at the statistical level *p ≤* 0.05, as measured using Duncan’s multiple range test.

**Table 4:** Effect of inoculating the growth medium with biocontrol agents on reducing the linear growth of *M. phaseolina*, *F. solani*, and *F. oxysporum* under *in vitro* conditions.

Biocontrol Agents	*M. phaseolina*		*F. solani*		*F. oxysporum*	
	Linear Growth (mm)	* Reduction (%)	Linear Growth (mm)	* Reduction (%)	Linear Growth (mm)	* Reduction (%)
*Bacillus subtilis*	28.5 ± 1.04 ^c^	68.3	25.7 ± 1.00 ^c^	71.4	27.9 ± 0.90 ^c^	69.0
*Gliocladium catenulatum*	24.1 ± 0.81 ^d^	73.2	21.5 ± 0.81 ^d^	76.1	23.9 ± 1.11 ^d^	73.4
*Trichoderma asperellum*	33.7 ± 1.31 ^b^	62.5	31.9 ± 1.01 ^b^	64.5	31.7 ± 1.02 ^b^	64.7
Positive control	11.5 ± 0.68 ^e^	87.2	9.1 ± 0.71 ^e^	89.8	9.5 ± 0.44 ^e^	89.4
Negative control	90.0 ± 0.0 ^a^	–	90.0 ± 0.0 ^a^	–	90.0 ± 0.0 ^a^	–

* The values of reduction were calculated based on the control values. Within the same column, values followed by the same letters are not significantly different at the statistical level *p ≤* 0.05, as measured using Duncan’s multiple range test.

**Table 5:** Effect of poisoning the growth medium with chemical inducers on reducing the linear growth of *M. phaseolina*, *F. solani*, and *F. oxysporum* under *in vitro* conditions.

Chemical Inducers	Conc. (ppm)	*M. phaseolina*		*F. solani*		*F. oxysporum*	
		Linear Growth (mm)	* Reduction (%)	Linear Growth (mm)	* Reduction (%)	Linear Growth (mm)	* Reduction (%)
Ascorbic acid	500	75.2 ± 1.60 ^b^	16.4	71.3 ± 1.65 ^b^	20.7	77.1 ± 1.42 ^b^	14.3
	1000	58.2 ± 1.46 ^d^	35.3	53.8 ± 1.17 ^d^	40.2	54.7 ± 1.23 ^d^	39.2
	2000	47.5 ± 1.83 ^f^	47.2	45.0 ± 1.19 ^f^	50.0	46.2 ± 1.42 ^f^	48.6
Potassium silicate	500	58.4 ± 1.79 ^d^	35.1	61.2 ± 1.65 ^c^	32.0	54.5 ± 1.39 ^d^	39.4
	1000	44.5 ± 1.14 ^g^	50.5	44.7 ± 1.17 ^f^	50.3	44.5 ± 1.31 ^g^	50.5
	2000	35.3 ± 1.14 ^j^	60.7	33.9 ± 1.19 ^i^	62.3	35.7 ± 1.30 ^i^	60.3
Salicylic acid	500	62.5 ± 1.65 ^c^	30.5	61.8 ± 1.44 ^c^	31.3	65.4 ± 1.60 ^c^	27.3
	1000	49.7 ± 1.50 ^e^	44.7	48.6 ± 1.04 ^e^	46.0	48.2 ± 1.55 ^e^	46.4
	2000	42.2 ± 1.41 ^h^	53.1	39.8 ± 1.20 ^g^	55.7	40.5 ± 1.20 ^h^	53.0
Positive control	500	40.3 ± 1.47 ^i^	55.2	36.5 ± 1.22 ^h^	59.4	43.1 ± 1.33 ^gh^	52.1
	1000	19.7 ± 1.12 ^k^	78.1	16.3 ± 1.15 ^j^	81.8	21.3 ± 1.20 ^j^	76.3
	2000	10.5 ± 1.08 ^l^	88.3	9.1 ± 1.17 ^k^	89.8	10.7 ± 1.19 ^k^	88.1
Negative control	–	90.0 ± 0.0 ^a^	–	90.0 ± 0.0 ^a^	–	90.0 ± 0.0 ^a^	–

* The values of reduction were calculated based on the control values. Within the same column, values followed by the same letters are not significantly different at the statistical level *p ≤* 0.05, as measured using Duncan’s multiple range test.

**Table 6:** Effect of biotic and abiotic inducers on controlling the root rot and wilt of roselle grown in soil pre-inoculated with *M. phaseolina*, *F. solani*, and *F. oxysporum* in a greenhouse.

Biotic/Abiotic Inducers	Rate Used	* Average Disease Control %					
		*M. phaseolina*		*F. solani*		*F. oxysporum*	
		Damping-Off %	Root Rot %	Damping-Off %	Root Rot %	Damping-Off %	Wilt %
		Biocontrol Agents (CFU mL^−1^)					
*Bacillus subtilis*	1 × 10^5^	34.8 ± 1.83 ^g^	35.7 ± 1.37 ^g^	33.4 ± 1.44 ^g^	35.0 ± 1.91 ^g^	33.9 ± 1.28 ^f^	35.9 ± 1.39 ^g^
	1 × 10^7^	47.3 ± 0.80 ^e^	50.0 ± 1.39 ^e^	52.7 ± 1.73 ^e^	56.4 ± 1.82 ^d^	52.9 ± 1.64 ^d^	59.5 ± 1.14 ^d^
	1 × 10^9^	65.0 ± 1.08 ^b^	70.0 ± 1.49 ^b^	70.1 ± 1.88 ^b^	68.2 ± 1.53 ^b^	70.0 ± 1.89 ^b^	75.2 ± 1.06 ^b^
*Gliocladium* *catenulatum*	1 × 10^5^	47.3 ± 1.59 ^e^	50.0 ± 1.75 ^e^	50.3 ± 2.24 ^e^	49.3 ± 1.69 ^e^	46.6 ± 1.88 ^e^	52.6 ± 1.39 ^e^
	1 × 10^7^	53.5 ± 1.33 ^d^	56.7 ± 1.46 ^d^	57.7 ± 2.00 ^d^	56.4 ± 1.82 ^d^	52.9 ± 1.92 ^d^	59.5 ± 1.24 ^d^
	1 × 10^9^	72.3 ± 1.89 ^a^	75.7 ± 1.37 ^a^	78.0 ± 1.92 ^a^	73.7 ± 1.64 ^a^	75.3 ± 1.33 ^a^	78.2 ± 1.06 ^a^
*Trichoderma asperellum*	1 × 10^5^	26.2 ± 1.73 ^h^	28.3 ± 1.10 ^h^	25.5 ± 1.45 ^h^	20.7 ± 1.71 ^h^	21.2 ± 1.69 ^g^	25.1 ± 1.07 ^h^
	1 × 10^7^	38.8 ± 0.92 ^f^	41.7 ± 1.77 ^f^	45.9 ± 1.34 ^f^	42.1 ± 1.64 ^f^	46.6 ± 1.96 ^e^	50.6 ± 1.45 ^f^
	1 × 10^9^	57.5 ± 1.18 ^c^	61.7 ± 1.61 ^c^	62.2 ± 1.15 ^c^	63.6 ± 1.88 ^c^	59.3 ± 1.78 ^c^	63.4 ± 1.23 ^c^
		Chemical Inducers (g/L)					
Ascorbic acid	1.0	16.0 ± 0.94 ^g^	20.5 ± 0.99 ^g^	23.5 ± 1.11 ^i^	19.6 ± 0.99 ^h^	13.2 ± 1.58 ^h^	24.9 ± 1.74 ^i^
	2.0	28.5 ± 0.73 ^f^	32.0 ± 0.94 ^f^	35.4 ± 1.51 ^g^	34.1 ± 0.95 ^f^	25.9 ± 1.54 ^f^	37.9 ± 1.61 ^f^
	4.0	55.5 ± 1.03 ^c^	60.1 ± 1.06 ^c^	58.2 ± 1.34 ^c^	63.2 ± 1.61 ^c^	51.3 ± 1.51 ^c^	66.0 ± 3.81 ^c^
Potassium silicate	1.0	28.5 ± 0.86 ^f^	38.8 ± 1.51 ^e^	36.4 ± 0.60 ^f^	34.1 ± 1.65 ^f^	26.9 ± 1.95 ^f^	35.9 ± 1.06 ^g^
	2.0	53.5 ± 1.21 ^d^	59.1 ± 1.11 ^c^	55.7 ± 0.84 ^d^	55.9 ± 1.51 ^d^	44.9 ± 1.43 ^d^	57.9 ± 1.50 ^d^
	4.0	68.0 ± 0.86 ^a^	73.7 ± 1.31 ^a^	72.1 ± 0.79 ^a^	65.0 ± 1.18 ^a^	64.0 ± 1.37 ^a^	75.0 ± 1.74 ^a^
Salicylic acid	1.0	28.5 ± 1.25 ^f^	32.0 ± 1.14 ^f^	29.9 ± 1.16 ^h^	26.9 ± 1.20 ^g^	19.5 ± 0.91 ^g^	31.9 ± 1.46 ^h^
	2.0	41.0 ± 1.73 ^e^	45.6 ± 1.19 ^d^	42.9 ± 0.95 ^e^	41.4 ± 1.40 ^e^	32.2 ± 1.06 ^e^	40.9 ± 1.46 ^e^
	4.0	60.8 ± 1.49 ^b^	65.9 ± 1.40 ^b^	61.3 ± 1.15 ^b^	63.5 ± 1.59 ^b^	57.6 ± 0.78 ^b^	69.0 ± 2.18 ^b^
		Biofertilizers (g/kg seeds)					
Mycorrhizeen	10	54.5 ± 1.40 ^c^	61.3 ± 1.00 ^c^	44.1 ± 1.20 ^c^	56.3 ± 1.50 ^c^	49.2 ± 0.70 ^c^	53.4 ± 1.90 ^c^
Microbein	10	35.2 ± 1.20 ^d^	41.7 ± 2.00 ^d^	26.9 ± 1.40 ^d^	38.2 ± 0.90 ^d^	31.0 ± 1.60 ^d^	32.5 ± 1.50 ^d^
Mycor.+ Micr.	10	59.2 ± 1.10 ^b^	68.0 ± 1.10 ^b^	52.5 ± 1.20 ^b^	64.4 ± 1.90 ^b^	55.4 ± 1.20 ^b^	59.2 ± 0.90 ^b^
Positive control	3	80.2 ± 0.50 ^a^	83.4 ± 1.70 ^a^	83.0 ± 0.90 ^a^	87.1 ± 1.20 ^a^	80.4 ± 1.50 ^a^	85.5 ± 2.40 ^a^

* The disease control values were calculated based on the control values. Within the same column, values followed by the same letters are not significantly different at the statistical level *p ≤* 0.05, as measured using Duncan’s multiple range test.

**Table 7:** Effect of treatments used alone or in combination on controlling root rot and wilt diseases of roselle in the field during the 2019 and 2020 growing seasons.

Treatments	Rate Used	* Average Disease Control %			
		Damping-Off %		Root Rot %	Wilt %
		Pre- Emergence %	Post- Emergence %		
*G. catenulatum* (Gc)	1 × 10^9^ CFU mL^−1^	73.2 ± 1.57 ^c^	60.4 ± 1.86 ^d^	64.2 ± 1.77 ^d^	70.4 ± 1.46 ^c^
*B. subtilis* (Bs)	1 × 10^9^ CFU mL^−1^	54.4 ± 1.61 ^f^	43.3 ± 1.08 ^g^	45.3 ± 1.00 ^g^	52.3 ± 1.57 ^f^
*T. asperellum* (Ta)	1 × 10^9^ CFU mL^−1^	44.1 ± 1.11 ^h^	35.4 ± 0.83 ^i^	34.1 ± 1.94 ^i^	39.6 ± 1.79 ^h^
Mixture of Gc + Bs + Ta	1 × 10^9^ CFU mL^−1^	79.2 ± 0.95 ^b^	72.5 ± 1.28 ^b^	75.6 ± 1.62 ^b^	77.2 ± 2.68 ^b^
Ascorbic acid (AA)	4 g L^−1^	39.1 ± 1.06 ^i^	30.0 ± 1.32 ^j^	28.3 ± 1.41 ^j^	32.5 ± 1.45 ^i^
Potassium silicate (PS)	4 g L^−1^	67.2 ± 1.70 ^d^	56.4 ± 1.06 ^e^	59.9 ± 1.79 ^e^	66.0 ± 1.67 ^d^
Salicylic acid (SA)	4 g L^−1^	49.4 ± 1.94 ^g^	41.2 ± 1.15 ^h^	42.5 ± 2.33 ^h^	45.0 ± 1.52 ^g^
Mixture of AA + PS + SA	4 g L^−1^	77.0 ± 2.06 ^b^	68.0 ± 1.35 ^c^	70.4 ± 1.23 ^c^	73.2 ± 1.00 ^b^
Mixture of Mycor. + Micr.	10 g kg^−1^ seeds	59.6 ± 1.93 ^e^	50.1 ± 1.05 ^f^	53.4 ± 1.55 ^f^	59.5 ± 1.62 ^e^
Positive control	3 g kg^−1^ seeds	89.0 ± 1.42 ^a^	80.4 ± 0.95 ^a^	85.2 ± 1.63 ^a^	83.7 ± 2.17 ^a^

* The disease control values were calculated based on the control values. The data collected are the mean of two repeated experiments during the 2019 and 2020 seasons. Within the same column, values followed by the same letters are not significantly different at the statistical level *p ≤* 0.05, as measured using Duncan’s multiple range test.

## Data Availability

Not applicable.
